# Fluvoxamine Ameliorates the Damage to the Neuro-Behavioral Status of Rats Caused by the Administration of Valproic Acid by Preventing Cognitive Memory Deficits and Decreased Hippocampal Cellular Proliferation

**DOI:** 10.7759/cureus.58578

**Published:** 2024-04-19

**Authors:** Maha Elbeltagy, Shahd Mansour, Jana A Zayed, Mohd Alqassam B Alrafayia, Ahmad Alhesa, Ahmed Salman

**Affiliations:** 1 Anatomy, University of Jordan, Amman, JOR; 2 School of Medicine, University of Jordan, Amman, JOR; 3 Pathology, University of Jordan, Amman, JOR

**Keywords:** antioxidant, cognitive memory, rodent model, valproic acid, spatial memory, hippocampal neurogenesis, fluvoxamine

## Abstract

Fluvoxamine is a major antidepressant of the selective serotonin-reuptake inhibitor class, previously studied as a drug that improves cognitive memory by enhancing hippocampal cell division and proliferation. Valproic acid (VPA) is a commonly used antiepileptic drug and mood stabilizer that has negative effects on cognitive memory as it inhibits cellular division and proliferation in the hippocampus. This study assessed the protective effects of fluvoxamine treatment versus the memory impairment, decreased hippocampal cellular proliferation, and weight loss produced by VPA treatment. The cognitive memory of 40 male Sprague-Dawley rats was assessed by the novel object location (NOL) test. Immunostaining by Ki67 and glutathione peroxidase 1 (GPX-1) was performed to quantify the number of dividing cells in the subgranular zone (SGZ) of the dentate gyrus and to assess the antioxidant activity of different treatments, respectively. Results showed that the VPA group had fewer Ki67-positive cells than the control group (p < 0.001), indicating reduced hippocampal proliferation. In contrast, the VPA and fluvoxamine combination group showed increased proliferation (p < 0.001) compared to VPA alone. Notably, fluvoxamine treatment significantly differed in cell counts compared to other groups (p < 0.001). Fluvoxamine also attenuated the weight loss caused by VPA (p < 0.0001). Our data suggested that fluvoxamine therapy attenuated the VPA-induced decrease in SGZ cellular proliferation, memory, and weight in rats.

## Introduction

Valproic acid (VPA) is widely used in the treatment of many neuropsychiatric disorders such as bipolar disorder, epilepsy, migraines, and behavioral disorders [1]. It has been shown that VPA treatment can bring about cognitive decline by inhibiting histone deacetylase enzymes; this causes hyperacetylation of DNA, leading to increased expression of growth arrest and pro-differentiation genes, thus blocking cellular proliferation [2]. VPA inhibits cell proliferation in vitro and in vivo by upregulating p21WAF1/CIP1, a cyclin-dependent kinase inhibitor, bringing about an increase in apoptosis [2]. Several studies have reported mild-to-moderate impairment in cognition and memory in adults taking VPA [3, 4]. Sub-chronic VPA therapy affects spatial memory and reduces cell proliferation in the subgranular zone (SGZ) of the dentate gyrus, while cessation of VPA is reportedly linked to improvements in cognition [2].

In the mammalian brain, neurogenesis is restricted to the SGZ of the dentate gyrus in the hippocampus and the SVZ of the lateral ventricles. Adult hippocampal neurogenesis contributes to learning and memory, such as episodic memory and spatial memory; this is supported by the finding that rat adult hippocampal neurogenesis (AHN) could be stimulated by learning spatial tasks [5].

The most important low-weight antioxidant synthesized in cells is glutathione (GSH). It is synthesized by adding cysteine to glutamate and then glycine in sequential order [6]. GSH is the primary antioxidant that buffers free radicals in brain tissue and protects cells from oxidative damage by reducing disulfide groups of cellular molecules or by scavenging free radicals and reactive oxygen species (ROS) [7]. Normal GSH levels are critical for spatial memory acquisition. GSH depletion induces failures in hippocampal synaptic plasticity mechanisms, which may be linked to spatial memory loss [8]. VPA causes oxidative stress by depleting GSH in neuronal tissue, which may lead to cell death in brain cells [7].

Fluvoxamine, a selective serotonin reuptake inhibitor (SSRI), is one of the most potent sigma-1 receptor agonists in its class. Sigma-1 receptor agonists demonstrate significant neuroprotective effects in various experimental models, and fluvoxamine has shown ameliorating effects in animal models of psychosis, depression, stress, anxiety, obsessive-compulsive disorder (OCD), and aggression. It has also been shown to reverse cognitive impairments. Fluvoxamine was discovered to increase nerve growth factor (NGF)-induced neurite outgrowth in PC12 cells via sigma-1 receptor agonism [9, 10].

To the best of our knowledge, there are no studies that investigated the effect of concomitant use of fluvoxamine and VPA on the cognitive impairment caused by VPA. However, one animal study demonstrated that fluoxetine, another SSRI, might prevent the decline in cognition due to VPA in rats [11]. In this study, we hypothesized that concomitant fluvoxamine and VPA administration can alleviate the cognitive impairment caused by VPA. Thus, this study aimed to investigate whether the concomitant use of fluvoxamine and VPA can decrease the cognitive impairment caused by VPA in rats. 

## Materials and methods

Animals

This study was carried out on* *40 male Sprague-Dawley rats that were two months of age and weighed 109-200 g. The rats were procured from the University of Jordan’s animal office in Amman, Jordan. Rats were weighed every other day from the day they arrived, and they were acclimatized to the lab conditions for two weeks before the experiment commenced. They were housed under standardized lab conditions: approximately 12 h light and 12 h dark cycle with *ad libitum* feeding and water, without husbandry conditions. The experiments were carried out between 8.00 h and 15.00 h Greenwich Mean Time (GMT). The protocol of this animal study was approved by the Scientific Research Ethics Committee of the University of Jordan with protocol number 2021-25. 

**Figure 1:**
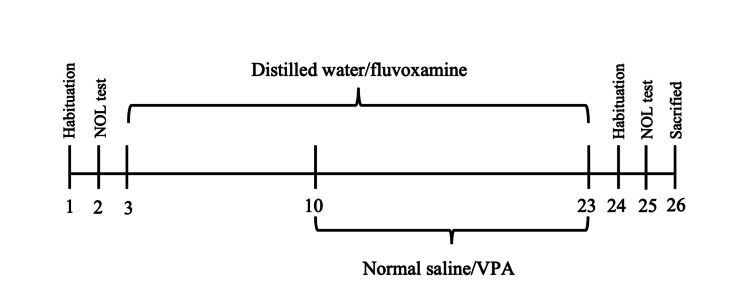
The drug administration protocol and behavioral testing timeline. The periods during which fluvoxamine and VPA were administered via feeding and injection, respectively, are represented by brackets. Rats were euthanized and their brains were removed one day after the final NOL behavioral testing. VPA: valproic acid NOL: novel object location

Rats were assigned to four groups randomly with 10 rats per group: (1) control group: rats received distilled water and normal saline (n = 10); (2) fluvoxamine group: rats received fluvoxamine (n = 10); (3) VPA Group:rats received VPA (n = 10); (4) fluvoxamine and VPA group: rats received fluvoxamine and VPA (n = 10). Figure 1 shows the drug administration protocol and behavioral testing timeline. 

Drugs and treatment schedule

VPA (Sanofi, Paris, France) and fluvoxamine (Abbott India, Mumbai, India) solutions were prepared immediately prior to the experiment. First, 1 mg fluvoxamine/ 100 g rat’s weight was dissolved in each rat’s daily portion of drinking water at 12.00 h GMT each day for 21 days from day 3-23. Also, 0.3 mg of VPA per 100 g rat’s weight was dissolved in normal saline 0.9% (1 ml/kg) and given as anintraperitoneal injection at 9.00 and 14.00 h GMT twice daily for 14 days [2, 12] from Days 10-23.

Behavioral testing

The novel object location (NOL) test [13], modified first by Dix and Aggleton and then by Umka et al. [2, 14], was used to assess the rats’ cognitive memory. The test set-up consisted of one semi-transparent box (49 cm×66 cm×40 cm), and an identical pair of colored plastic cone-shaped toys (15 cm×7 cm diameter). Each toy was taped to the floor of the box 7 cm away from the corner, so the rats could move freely around the toy while it stayed in place. The experiments were recorded with a camcorder at an illumination of (350-400 lux) [11].

**Figure 2:**
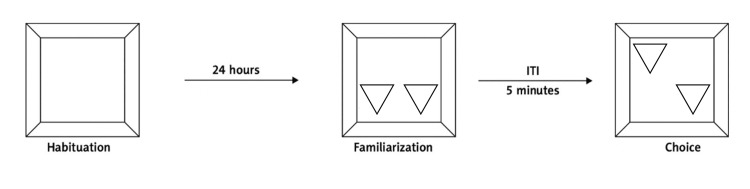
The object location recognition test protocol spanned two days. Initially, animals were given one hour to acclimate to the test arena on the first day. On the subsequent day, two identical objects were positioned in different locations within the box, allowing the animal to explore for three minutes (familiarization trial). Animals were removed from the box for a five-minute inter-trial interval (ITI), after which they were reintroduced to the box with the objects' locations altered for three minutes (choice trial).

The test procedure was carried out two days before, and one day after the drug administration. It consisted of habituation, familiarization, and choice trials (NOL test as shown in Figure 2). During the habituation day, the objects were removed, and each rat freely explored the box for 60 minutes. After that, the rat was removed from the box and returned to its original cage for 24 hours before the familiarization trial. In the familiarization trial, two identical objects were each put on opposite corners of the box, and the rat was allowed to explore the toys for three minutes. Then, it was returned to its holding cage for five minutes after which the toys were removed from the box and cleaned with 76% ethanol to remove any olfactory signals. In the choice trial, one of the objects was returned to its original location while the other was placed behind its original position. The rat was allowed to explore the toys for three minutes and then was returned to its original cage. To ensure that there were no disruptions during the trials, no one was allowed near the room where the tests were being held. Once the rat directed its nose in the direction of the objects at less than 2 cm and either actively sniffed, licked, chewed, or moved its vibrissae, exploratory activity was recorded [14] (Figure 2).

Histology and immunohistochemistry

Ki67 and glutathione peroxidase 1 (GPX1) immunohistochemistry was used to quantify the number of proliferating cells and the antioxidant activity present in the SGZ of the dentate gyrus after euthanasia. After behavioral testing, rats were euthanized by ether inhalation, and their brains were extracted and trimmed, then fixed in 3% glutaraldehyde overnight*. *The following day, the brains blocked in paraffin were sectioned at 4-micrometer-thickness on a Leica microtome (LEICA RM2235 Microtome, Leica Biosystems Nussloch, Nussloch, Germany) and placed on slides that were positively charged. These slides were utilized for staining with hematoxylin and eosin (routine) and then for immunohistochemical analysis with Ki67 and GPX1. The paraffin sections were dewaxedwith* *xylene*, *rehydrated with a series of graded ethanol aqueous solutions, and then rinsed in deionized water. Epitope retrieval for Ki67 was carried out with a 95°C water bath for 60 minutes in Tris/EDTA solution (0.01 M, pH 9.0). For GPX1, the paraffin sections were retrieved in a 95°C water bath for 25 minutes in sodium citrate solution (0.01 mM, pH 6.0). Following rinsing with deionized water, paraffin sections were treated with 3% hydrogen peroxide for 10 minutes at room temperature and then washed thoroughly in phosphate buffer solution (PBS, 0.1 M, pH 7.4). Using 5% bovine serum albumin (Atlas Medical, Berlin, Germany), non-specific immunoglobulin binding was blocked in PBS for 60 minutes at room temperature. After the blocking step, paraffin sections were incubated with GPX1 antibody (rabbit polyclonal, 1:750 dilutions, PA5-95206, Invitrogen, Thermo Fisher Scientific, Waltham, MA) and Ki67 antibody (mouse monoclonal, 1:100 dilution, ab279653, Abcam, Cambridge, UK) overnight at 4°C. The primary antibody was diluted in PBS containing 0.2% Tween 20 Detergent (Tween 20 viscous liquid, CAS 9005-64-5, Sigma-Aldrich, St. Louis, MO). Afterward, paraffin sections were washed twice with PBS for five minutes and incubated with complement reagent (ab236466, Abcam, UK) for 10 minutes at room temperature. Next, paraffin sections were rinsed in PBS for five minutes and incubated with goat anti-rabbit linker (ab236466, Abcam, UK) for 15 minutes at room temperature. The paraffin sections were washed twice with PBS and incubated with 3,3′-diaminobenzidine (DAB) for six minutes at room temperature for color development. The paraffin sections were counterstained with hematoxylin for five minutes. Finally, the slides were dehydrated with an ascending series of ethanol aqueous solutions, cleared with fresh xylene, and cover-slipped with dibutylphthalatepolystyrene xylene (DPX) mounting media.

The appropriate positive and negative control slides were included in each staining run and maintained for quality control. For Ki67 and GPX1, rat intestinal epithelium tissue sections were used as positive controls. Negative controls were performed by omitting the primary antibody and replacing it with PBS. The dark brown cytoplasmic pattern shows positive staining for GPX1 while a dark brown nuclear pattern shows positive staining for Ki67.

Statistical analysis

Statistical analysis was done with p<0.05 considered to be statistically significant, and graphs were created using GraphPad Prism 5.0 (GraphPad Software Inc., Boston, MA). The new time spent by the rats on exploring the novel object was divided by the old and new exploration times to calculate the preference index (PI) which was compared among the different groups. To compare the exploration times for rats in each group in the NOL choice trials, Student's paired t-tests were used. As for the Ki67-positive proliferating cells and the preference ratios, a one-way ANOVA with Bonferroni's post-test was used for the comparison between groups. A two-way ANOVA with Bonferroni's post-test was utilized for the comparison of replicate means of the rats' weights during the period of injection between the groups. 

## Results

The effect of treatment on recognition of the novel object location task

**Figure 3:**
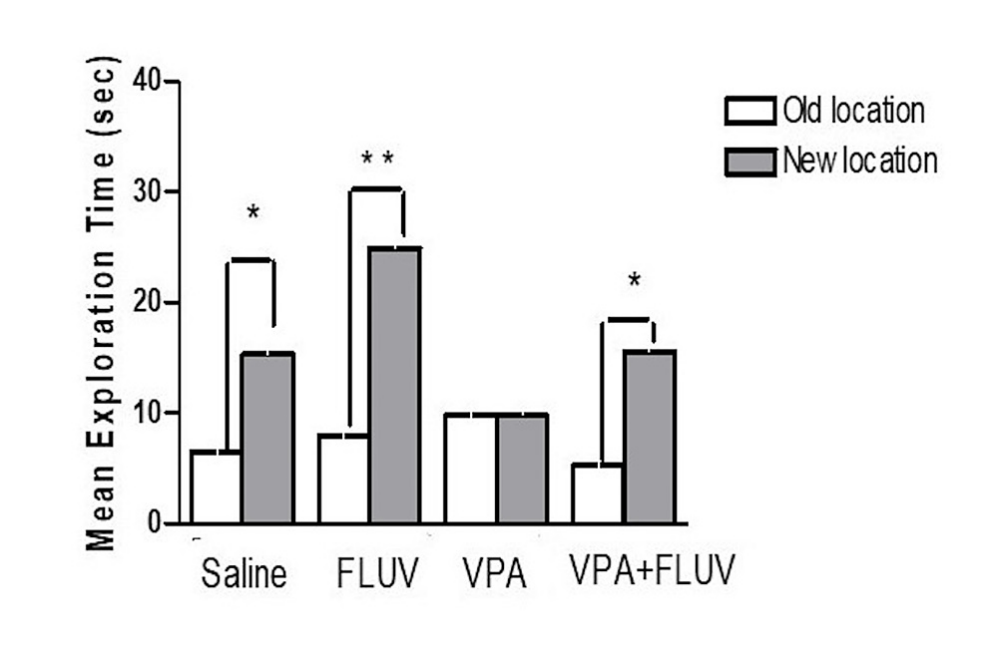
Effect of different treatments on the NOL task performance (choice trial). Saline-injected controls explored the novel object significantly more than the old one (p <0.05). Animals treated with fluvoxamine significantly explored the novel location more than the old one (p <0.01). Animals treated with VPA failed to differentiate between the two locations (p=0.9). Co-administration of fluvoxamine and VPA significantly improved the spatial working memory of the animals (p <0.01). NOL: novel object location; VPA: valproic acid; FLUV: fluvoxamine

Results showed that rats from all the groups were able to perform the NOL test prior to drug administration as the time they spent exploring the two identical objects (which were placed in different spots during the familiarization and the choice trials) was nearly equal (Figure 3). During the choice trial after treatment, the animals in the control, fluvoxamine only, and fluvoxamine and VPA groups spent significantly more time exploring the object that was moved to a new site in comparison to the object placed in the old location (p < 0.01 for fluvoxamine only, and fluvoxamine and VPA, p < 0.05 for control) (Figure 3). However, the group that received VPA only demonstrated no significant difference in time spent exploring the objects, whether they were placed in a novel or familiar location (p = 0.9) (Figure 3). 

**Figure 4:**
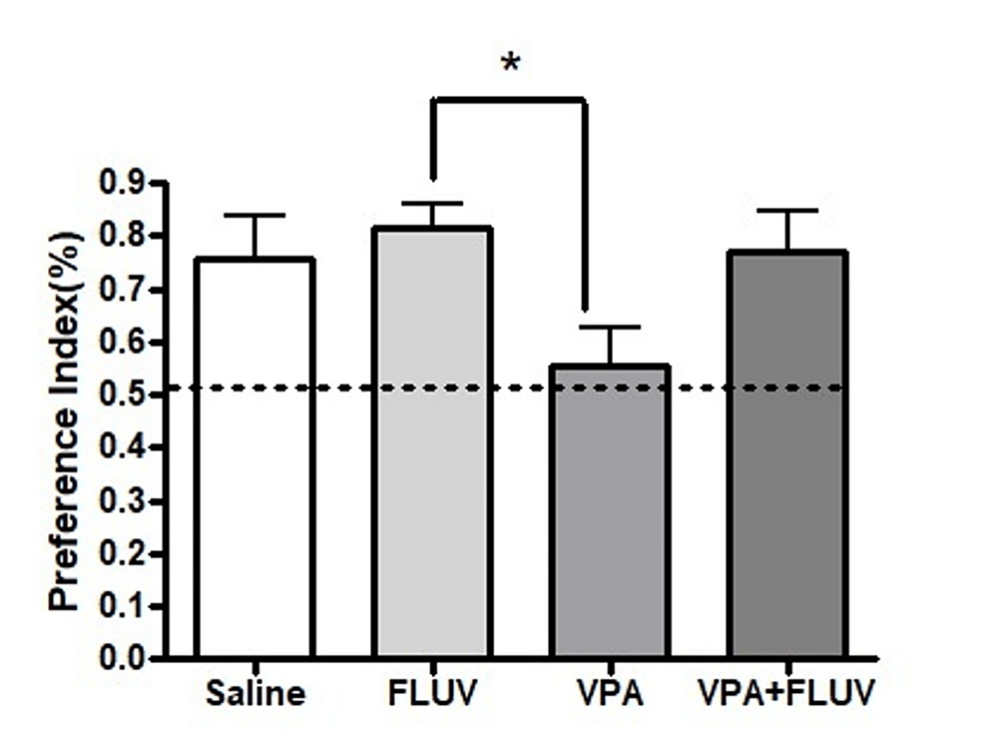
PI of different treatments. Animals treated with VPA had significantly lower preference index compared to both control and fluvoxamine treated (p <0.05). The analysis was done using one-way ANOVA with Bonferroni post-test. PI: Preference index; VPA: valproic acid; ANOVA: analysis of variance; FLUV: fluvoxamine

A significant reduction in PI was found in the fluvoxamine-treated group versus the VPA-treated group (p < 0.0) (Figure 4). 

The effect of treatment on proliferating cell counts

**Figure 5:**
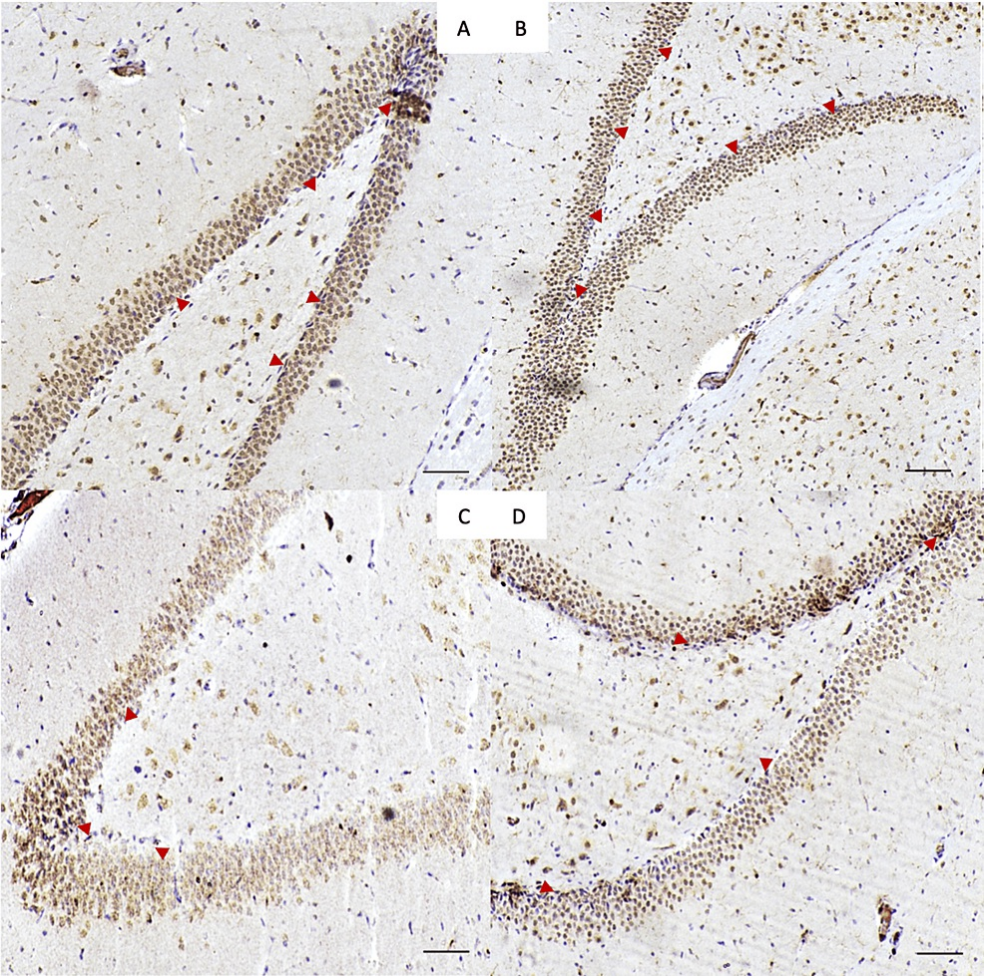
Representative immunohistochemistry photos taken from the dentate gyrus of (A) control group, (B) fluvoxamine group, (C) VPA group, and (D) fluvoxamine with VPA group. Ki67-positive cells (arrows) appear dark, indicating proliferation.

**Figure 6:**
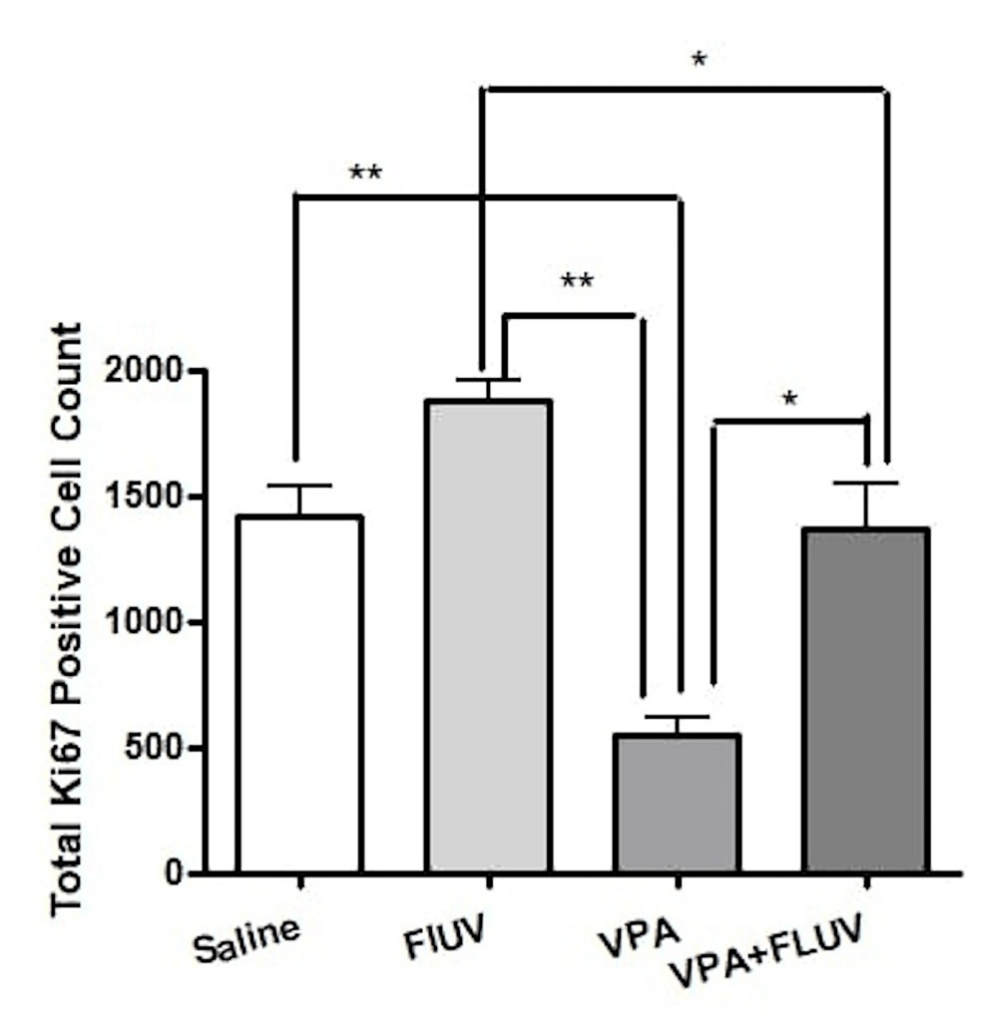
Effect of different treatments on total Ki67-positive cell counts. Animals treated with VPA had significantly lower total Ki67-positive cell counts compared to the control (p < 0.001). Conversely, the combined VPA and fluvoxamine group showed increased hippocampal proliferation compared to the VPA group (p < 0.001). Lastly, VPA combined with fluvoxamine had lower cellular proliferation than fluvoxamine alone (p < 0.05). VPA: valproic acid

To quantify the number of cells dividing in the dentate gyrus SGZ, immunostaining with Ki67 was done which revealed a significant difference in the number of Ki67-positive cells between the four groups (p < 0.0001) (Figure 5). In the VPA-treated group, there was a significant reduction in Ki67-positive cells when compared to the control group* *(p < 0.001). Conversely, the combined VPA and fluvoxamine group showed more active hippocampal cell proliferation as opposed to the VPA group* *(p < 0.001). Lastly, VPA combined with fluvoxamine had lower cellular proliferation than fluvoxamine alone (p < 0.05). Fluvoxamine has demonstrated a significant difference in proliferating cell counts when compared to all other groups, with a significant difference between VPA and fluvoxamine versus fluvoxamine treatment alone (p < 0.001) (Figure 6).

The effect of treatment on antioxidant activity

**Figure 7:**
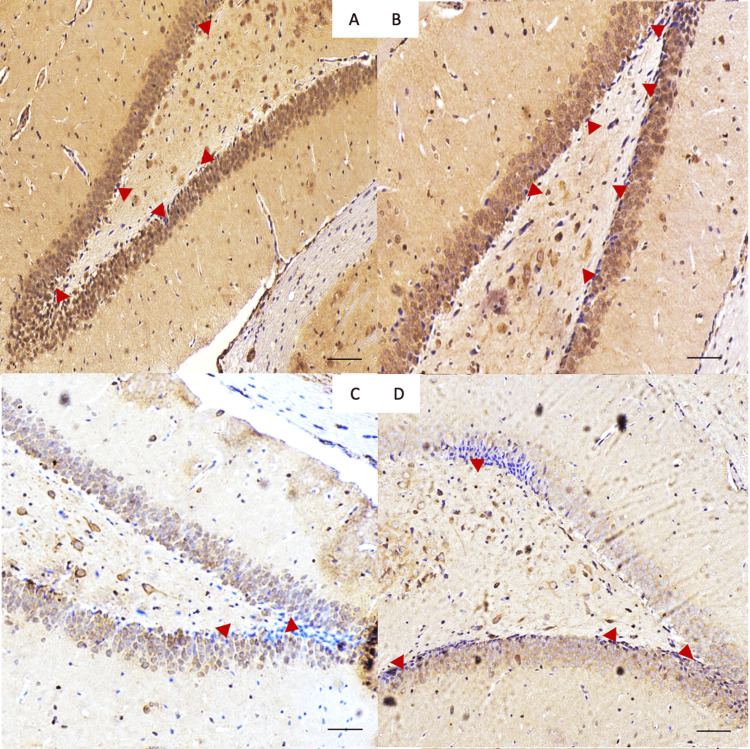
Representative immunohistochemistry photos taken from the dentate gyrus of (A) control group, (B) fluvoxamine group, (C) VPA group (D) fluvoxamine with VPA group. Glutathione-positive cells (arrows) appear dark, indicating proliferation.

**Figure 8:**
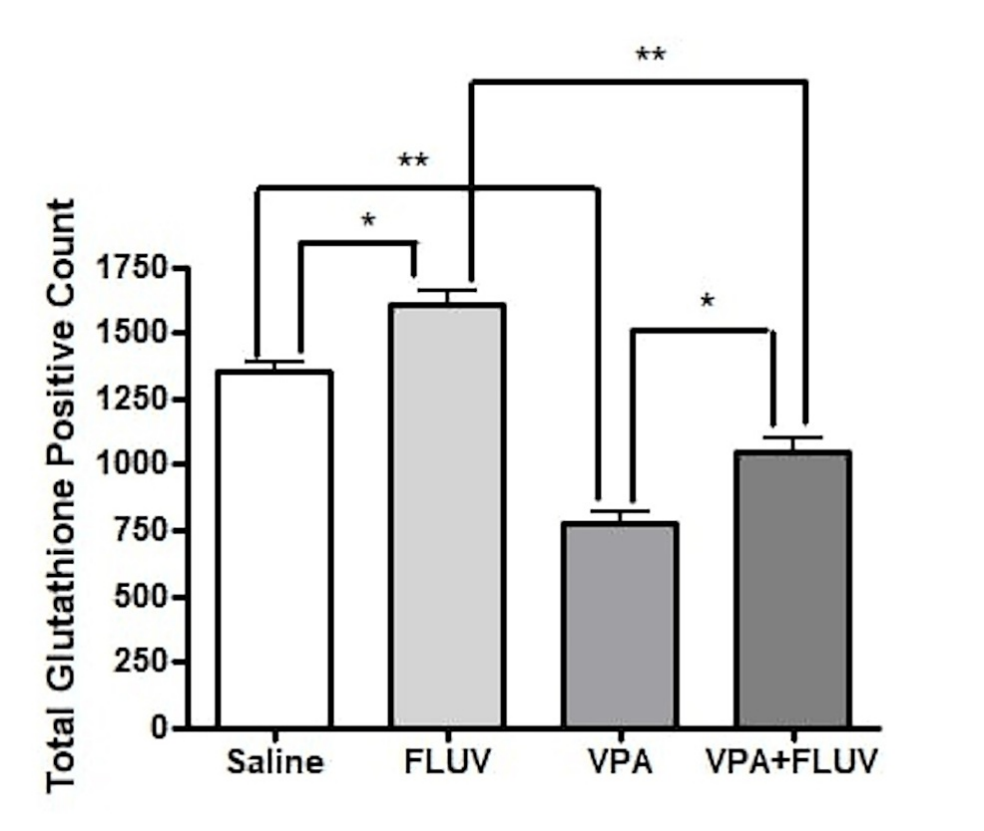
Effect of different treatments on total glutathione positive counts. There was a significant difference between positively stained cell numbers between control and VPA only, as well as control and combined VPA and fluvoxamine-treated cells (p < 0.001) for both. As for control versus fluvoxamine-treated cells, there was also a significant difference in positively stained cells (p < 0.01). When comparing cells treated with VPA and those with fluvoxamine, there is a significant difference (p < 0.001) with much fewer proliferating cells in animals treated with VPA. VPA: valproic acid

Immunostaining with GPX1 was done to assess the antioxidant activity of different treatments (Figure 7). There was a significant difference between positively stained cell numbers between control and VPA only, as well as control and combined VPA and fluvoxamine-treated cells (p < 0.001) for both. As for control versus fluvoxamine-treated cells, there was also a significant difference in positively stained cells (p < 0.01). When comparing cells treated with VPA and those with fluvoxamine, there is a significant difference (p < 0.001) with much fewer proliferating cells in animals treated with VPA (Figure 8).

The effect of treatment on the weight of the rats

**Figure 9:**
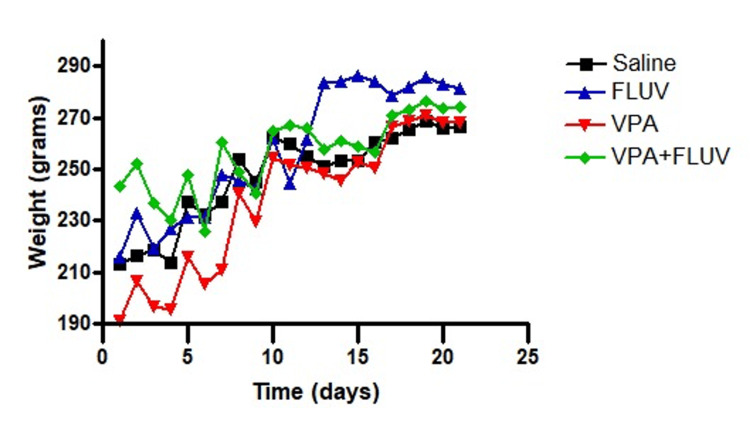
Effect of different treatments on the weight of animals. Significant differences were observed between groups as a result of treatment and injection days (p < 0.0001). Throughout the injection period, rats treated with VPA exhibited a notable reduction in weight compared to those treated with saline and fluvoxamine (p < 0.0001). The analysis utilized a two-way ANOVA with Bonferroni’s post-test for comparing replicate means, conducted using GraphPad Prism 4. VPA: valproic acid ANOVA: analysis of variance

There was a significant difference between the rats’ weights in different groups (p < 0.0001) (Figure 9). Overall, the weight of VPA-treated rats was significantly reduced compared to the rats treated with VPA and fluvoxamine (p < 0.0001). Fluvoxamine attenuated the loss of weight which was caused by VPA (p < 0.0001).

## Discussion

The current study aimed to investigate the effect of VPA on cognitive behavior and cellular proliferation for hippocampal neurogenesis using a rodent model. Furthermore, we aimed to determine if prior therapy with the antidepressant fluvoxamine would be protective against VPA's unfavorable behavioral and cellular consequences, including oxidative stress and neuronal damage caused by VPA. NOL testing, previously established as useful in measuring spatial memory dependent on the hippocampus, was used in this study [2, 15-17]. Our findings revealed that rats given VPA were unable to distinguish between two identical objects after one of them was transferred to a new position. On the other hand, rats that were treated using saline control or fluvoxamine alone demonstrated a normal ability to discriminate between the new object placement, as evidenced by significantly longer time spent exploring the object moved to the new location. To discriminate this way, a functional dentate gyrus is required, and the ability is lost if hippocampal neurogenesis is impaired [2, 15-17]. On the other hand, fluvoxamine has been shown to have neuroprotective capability by attenuating neurotoxin-induced-dopaminergic degeneration in an animal model of Parkinson’s disease [18]. Consequently, the co-administration of VPA and fluvoxamine to animals reduced the progression of impairment in the rats’ ability to discriminate between locations of objects. This wasvalidated by calculating the PI, which revealed that animals spent a longer period on the object placed in the novel location than would be predicted by chance, except the VPA-treated group*, *which spent an equal amount of time exploring objects located in the novel and familiar spots.This* *supports previous studies that revealed that VPA can induce mild to moderate cognitive deficits in patients [3, 19-21] and impair spatial memory in animals [2]. 

Spatial memory requires adequate hippocampal neurogenesis [22, 23]. VPA causes reduced cellular proliferation and ultimately causes spatial memory deficits [2]. On the contrary, SSRIs were shown to enhance spatial memory functions and cognition since they have neuroprotective properties, such as enhancing the proliferation of neural progenitor cells in the hippocampus [24]. This is also seen in a study with a rodent model, where fluvoxamine treatment significantly improved memory function as measured by a novel object recognition test [25]. The results of this study showed that fluvoxamine-treated animals had a higher number of proliferating cells in the SGZ when compared with rats treated with either VPA or saline only. When administered concomitantly with VPA, it was able to limit the reduced cognition and cellular proliferation found in animals treated with VPA solely. The duration of fluvoxamine administration in this study is similar to that used by Elbeltagy et al. [15] and it resulted in more dentate gyrus cellular proliferation* *[17, 26]. 

In a study on rodent animal models, SSRIs caused significant weight gain, partly due to increased carbohydrate preference. However, fluvoxamine was shown to cause the least weight gain among other SSRI agents due to its positive effect on the resting basal metabolic rate [27]. In comparison, weight gain is a well-known adverse effect of VPA treatment occurring in up to 71% of exposed patients, and it is the most common reason why patients choose to discontinue VPA treatment [28].

In our study, we noticed a significant reduction in weight in the VPA-injected rats as opposed to the control group and other groups during a period of time when injections were administered. This is in line with the literature, where it was shown in a randomized controlled trial done on healthy adult rodents that VPA was associated with a significant fall in body weight along with a failure to produce obesity in VPA-treated rats in comparison with saline [29]. In our study, fluvoxamine co-treatment effectively improved VPA-induced weight loss in rats. 

GSH has critical roles in protecting cells from oxidative damage and maintaining redox homeostasis, and it is the most abundant low molecular-weight thiol compound synthesized in cells [6]. The hippocampal dentate gyrus is a region of the brain that is responsible for learning and memory performance and is extremely sensitive to ROS-induced oxidative stress [30]. This oxidative stress can cause cellular damage and death, due to damage to vital cellular components such as lipids, proteins, and DNA [31]. In fact, oxidative damage to neuronal components underlies the molecular basis of neurodegeneration and brain aging. As such, normal GSH levels are crucial for the acquisition and maintenance of spatial memory [8]. However, VPA was found to induce oxidative stress by compromising the antioxidant status of the neuronal tissue, and this was demonstrated as a significant depletion in GSH [7]. In another study on oxidative stress due to lipopolysaccharide in liver tissue, this reduction in GSH was ameliorated after fluvoxamine treatment, and SSRIs were found to reduce oxidative stress in mice [32]. In our studies, the decrease in levels of GPX 1 produced by VPA was prevented by concomitant fluvoxamine treatment. 

Limitations

Using an animal model to test drug co-administration might have some drawbacks since animals and humans have varying metabolism, physiology, and genetics. This means that animal models might not consistently show how a drug, treatment, or disease will affect humans. Additionally, the controlled environments employed in this study do not represent real-world complexities.

## Conclusions

Treating rats with VPA produced a deficit in memory and a reduction in levels of hippocampal cellular proliferation and antioxidant activity, as well as a significant reduction in rats' weight overall. However, concomitant administration of fluvoxamine with VPA significantly opposed those effects, by promoting hippocampal cellular proliferation and antioxidant activity, enhancing rats' memory and spatial awareness, and remarkably ameliorating weight loss induced by VPA treatment. 
